# Evolution of Predicted Acid Resistance Mechanisms in the Extremely Acidophilic *Leptospirillum* Genus

**DOI:** 10.3390/genes11040389

**Published:** 2020-04-03

**Authors:** Eva Vergara, Gonzalo Neira, Carolina González, Diego Cortez, Mark Dopson, David S. Holmes

**Affiliations:** 1 Center for Bioinformatics and Genome Biology, Fundación Ciencia & Vida, Santiago 7780272, Chile; 2 Centro de Genómica y Bioinformática, Facultad de Ciencias, Universidad Mayor, Santiago 8580745, Chile; 3 Centre for Ecology and Evolution in Microbial Model Systems, Linnaeus University, SE-391 82 Kalmar, Sweden; 4 Universidad San Sebastian, Santiago 7510156, Chile

**Keywords:** *Nitrospira*, extreme acidophile, acid mine drainage (AMD), bioleaching, evolution, acid resistance, comparative genomics, horizontal gene transfer (HGT), phylogenetics

## Abstract

Organisms that thrive in extremely acidic environments (≤pH 3.5) are of widespread importance in industrial applications, environmental issues, and evolutionary studies. *Leptospirillum* spp. constitute the only extremely acidophilic microbes in the phylogenetically deep-rooted bacterial phylum Nitrospirae. Leptospirilli are Gram-negative, obligatory chemolithoautotrophic, aerobic, ferrous iron oxidizers. This paper predicts genes that Leptospirilli use to survive at low pH and infers their evolutionary trajectory. Phylogenetic and other bioinformatic approaches suggest that these genes can be classified into (i) “first line of defense”, involved in the prevention of the entry of protons into the cell, and (ii) neutralization or expulsion of protons that enter the cell. The first line of defense includes potassium transporters, predicted to form an inside positive membrane potential, spermidines, hopanoids, and Slps (starvation-inducible outer membrane proteins). The “second line of defense“ includes proton pumps and enzymes that consume protons. Maximum parsimony, clustering methods, and gene alignments are used to infer the evolutionary trajectory that potentially enabled the ancestral *Leptospirillum* to transition from a postulated circum-neutral pH environment to an extremely acidic one. The hypothesized trajectory includes gene gains/loss events driven extensively by horizontal gene transfer, gene duplications, gene mutations, and genomic rearrangements.

## 1. Introduction

Although microorganisms such as *Escherichia coli*, *Vibrio cholerae*, and *Salmonella* spp. are neutrophiles, some strains survive acid shock during transient passage through low pH conditions such as in the stomach (<pH 3.5). In addition, the neutrophile *Helicobacter pylori* survives and grows in the stomach by creating its own near neutral pH environment via the hydrolysis of urea to produce CO_2_ and NH_3_ that buffer the acidity of the local environment (reviewed in [1]). Genes and mechanisms that these microorganisms use to survive acid shock and the regulatory networks that control their expression were reviewed in [2,3,4,5,6]. Due to the transitory nature of the acid shock response, these neutrophiles have been dubbed “amateur” acidophiles [5]. In contrast, extreme or “professional” acidophiles are organisms that thrive in extremely low pH environments (≤pH 3.5). They include Bacteria, Archaea, and Eukarya and are found widely distributed across the Tree of Life (reviewed in [1,7,8,9,10]).

Extreme acidophiles maintain a cytoplasmic pH close to neutral while confronted with a >10^4^ fold proton gradient across the cytoplasmic membrane [1,8,11]. Extreme acidophiles use many of the same mechanisms to cope with acid resistance as the amateur acidophiles, but also have additional mechanisms that allow them to cope with greater proton gradients [1]. Extreme acidophiles have several “first line” of defense mechanisms against low pH that function both within and outside the cytoplasmic membrane to exclude protons from entering the cell. They also have second line of defense mechanisms that consume or expel protons that have penetrated the cytoplasmic membrane. Less is known regarding the acid response mechanisms of extreme acidophiles than their amateur counterparts and especially the evolutionary events that resulted in the ability to grow at very low pH values.

The ”first line” of defense of extreme acidophiles includes the development of an inside positive membrane potential (Donnan potential), such that there is a chemiosmotic barrier that inhibits positively charged protons from crossing the membrane and acidifying the cytoplasm. The membrane potential is likely generated by potassium ions (and to a lesser extent, sodium ions) accumulated via the Kch, Kdp, and Trk K^+^ channel proteins [12,13,14]. Kch is a member of the voltage-gated ion channel superfamily mostly studied in eukaryotes, wherein they play a role in membrane potential regulation. The KdpFABC complex is a potassium transporting P-type ATPase with a high affinity to potassium [15], while the products of three unlinked genes (*trk*AEH) are necessary for Trk activity in *E. coli* and similar enterics [16]. Evidence to support the role of potassium ions in forming this inside positive membrane potential includes the observation that removing potassium and/or sodium decreases the ability of *Sulfolobus* spp. [12,17] and *Acidithiobacillus thiooxidans* [18] to cope with acid resistance. All three of these potassium transporters have been previously reported in *Leptospirillum ferriphilum* as part of its pH homeostasis mechanism [13]. The internal positive membrane potential is a key difference between professional acidophiles compared to amateur acidophiles [19]. However, definitive proof of the role of potassium in generating the membrane potential is still lacking.

A second cytoplasmic membrane-associated method to reduce proton penetration into the cell involves the use of spermidine. Spermidine is a positively charged, aliphatic polycation polyamine that together with spermine, putrescine, and cadaverine are found in all three domains of life. These polyamines are involved in a wide range of biological functions including acid stress responses (reviewed in [3,20]). The mechanism by which spermidine protects against acid stress is not completely understood but it is reported that it stimulates RpoS (σ38) synthesis in *E. coli* that, in turn, regulates many genes involved in the acid stress response [21,22,23]. Spermidine has been detected in *Leptospirillum ferrooxidans* [24] and *spe*EH, potentially involved in its biosynthesis, and is also predicted in the *L. ferriphilum* genome [13]. Orthologs of *spe*EH have higher transcript numbers during acid stress in the extreme acidophile *Acidithiobacillus ferrooxidans*, an observation that is consistent with the idea that they may be involved in acid resistance [25].

Hopanoid membrane lipids have also been shown to be involved in the first line acid resistance response [26,27]. Hopanoids have diverse proposed functions including modulating membrane fluidity and permeability. They may also be involved in other stress responses including oxidative stress and high temperature [28,29,30]. Hopanoid (*hpn*) genes are often found in gene clusters (e.g., [31]) and code for enzymes that either synthesize hopanoids from squalene or modify them, yielding different hopanoid molecules that may have a specific stress related function in various strains [26]. Hopanoids are present in acidophiles [32,33], and their RNA transcripts are detected in acid mine drainage (AMD) [34]. Deletion of hopanoid synthesis genes in neutrophiles resulted in impaired growth at low pH [28,30].

The starvation-inducible outer membrane protein (Slp) has been implicated in acid resistance. For example, in enteropathogenic *E. coli*, *slp* is located in a gene cluster that has been associated with acid resistance [35]. Co-expression with other acid resistance genes of this cluster has been reported [36]. Although the details of the mechanism of action of Slp remain unknown [37], it is proposed that it prevents the entrance of organic acids such as succinate, lactate, and formate across the outer membrane [38]. Organic acids have been shown to be highly toxic to acidophiles [39].

Second line of defense mechanisms include proton antiporters such as NahP-/NhaA-type Na^+^/H^+^ exchangers. These proteins constitute a large family of integral membrane proteins with roles related to homeostasis of sodium in high salt environments and regulation of intracellular pH [40]. In addition, voltage gated ClC-type Cl^−^/H^+^ transporters [41] are essential for the survival of *E. coli* under extreme acid stress [42]. In addition to its proton exporting function, the ClC channel is also proposed to prevent inner-membrane hyperpolarization (inner positive) in *E. coli* at extremely acidic pH as a result of the action of other acid resistance systems such as arginine and glutamate decarboxylation systems [43]. A similar function for ClC in acid resistance has been proposed in the amateuracidophile *Bacillus coagulans* [44].

Another second line of defense mechanism is the use of amino acid decarboxylation systems such as the Gad glutamate decarboxylase system. These systems have two components: (i) an inner membrane amino acid antiporter that imports the amino acid in exchange for its decarboxylated form and (ii) a cytoplasmic decarboxylase that catalyzes the proton-consuming decarboxylation of an amino acid [45]. Glutamate decarboxylase is the major response in *E. coli* under extreme acid conditions [6]. H^+^-consuming reactions during acid stress have also been observed in *Acidithiobacillus caldus* [33]. Several amino acid decarboxylases genes are present in *Leptospirillum*, e.g., [13,46], and transcripts coding for glutamate, arginine, and lysine decarboxylation have been detected in an AMD community [34].

In this study, we predict and analyze both first and second line of defense acid resistance systems in the extremely acidophilic genus *Leptospirillum.* We create a global model of pH homeostasis mechanisms that suggests how this genus can survive in hyper-acidic conditions. We use parsimony to infer the evolutionary trajectories of the gene gains/losses and gene mutations that are hypothesized to be involved in acid resistance. The *Leptospirillum* genus was chosen as a model system for this study because it is one of the most extreme bacterial acidophiles known with a growth pH range between 0.7 and 2.2 [47,48]. In addition, fluorescent in situ hybridization and “omic” studies highlight *Leptospirillum* as one of the main active taxa in extremely low pH natural and man-made acid mine drainage environments [49,50,51,52,53,54,55,56,57,58] and in commercial copper and gold biorecovery operations [47,59].

## 2. Methods

### 2.1. Genomes and Quality Assessment

**Figure 1:**
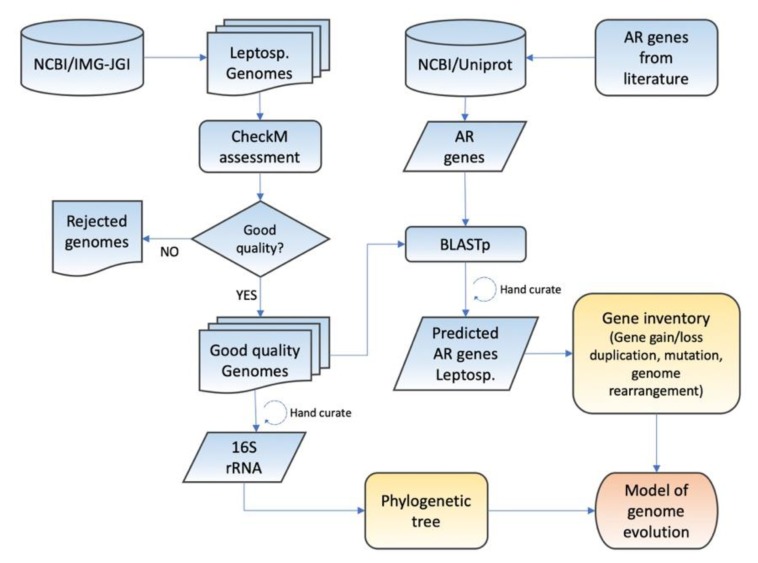
Bioinformatic pipeline outlining the strategy used for developing a model of the evolution of acid resistance (AR) mechanisms in the extremely acidophilic. Leptosp. = *Leptospirillum* genus.

Fifteen complete and partial genome sequences from the *Leptospirillum* genus were downloaded from the NCBI RefSeq genomic database, National Center for Biotechnology Information (NCBI), and Joint Genome Institute’s IMG-M databases (https://img.jgi.doe.gov/) in August 2018. In addition, the *Nitrospira marina* Nb-295 genome (IMG Genome ID 2596583682, https://img.jgi.doe.gov/) was downloaded as an outgroup for the *Leptospirillum* genus. Quality assessment of the 16 Nitrospirae genomes was carried out by CheckM [60], defining >90% base completeness and <10% contamination as high-quality genomes according to a bioinformatics pipeline (Figure 1).

### 2.2. Phylogenetic Analysis

16S rRNA gene sequences from organisms within the Nitrospirae phylum were identified by a BLASTn comparison against the SILVA [61], RDP [62], and GREENGENES rRNA databases [63] with an E-value threshold of 1E^−5^. Gene sequences with a minimum length of 1400 nucleotides were selected [64]. Alignments of 16S rRNA sequences were generated with MAFFT with the L-INSI iterative refinements option [65,66], MUSCLE [67], and T-Coffee alignment tools [68]. A maximum-likelihood tree was constructed with IQTREE [69], and the best-suited evolutionary models were selected using the model test tool implemented in IQTREE [70] according to the Bayesian (BIC) and Akaike information criterion (AIC). The robustness of the inferred tree was assessed using the nonparametric bootstrap procedure implemented in IQTREE (1000 replicates of the original datasets) with the ultrafast bootstrap option [71]. The final tree was visualized using Figtree (http://tree.bio.ed.ac.uk/software/figtree/).

### 2.3. Prediction of Mobile Genetic Elements and Genome Islands

Insertion elements and transposases were predicted and classified using TnpPred [72] and ISsaga [73,74]. Sigi-HMM [75] was used to predict genes obtained by Horizontal Gene Transfer (HGT). IslandViewer 4 [76] was used to predict genomic islands. Genome contexts of predicted acid resistance genes, associated hypothetical genes, and predicted mobile elements were analyzed using STRING [77] and by manual inspection using MAUVE [78] and Artemis [79].

### 2.4. Identification of Genes Related to Low pH Resistance

Genes reported to be involved in acid resistance were identified through an extensive literature search [4,5,6,38,80,81,82,83,84,85,86,87]. A search for similar genes in *Leptospirillum* and *Nitrospira marina* Nb-295 genomes was carried out through BLASTp comparison [88] using a minimal *E-*value cutoff of 1e^−5^. Synteny blocks and conservation of genetic context between *Nitrospiraceae* genomes were determined by MAUVE [78]. Genomic contexts were visualized by Artemis [79]. Conservation of sequences and domains within the *Leptospirillum* genus were analyzed and visualized by the WebLogo [89,90] and AliView [91] alignments tools. Selected *Nitrospiraceae* genes were compared against the UniProt and NCBI databases by BLASTp to identify orthologous proteins in other microorganisms. This collection of sequences was aligned with MAFFT using the L-INSI iterative refinements option [65,66]. IQTREE was used to construct a maximum-likelihood tree with 1000 replicates by the ultrafast bootstrap option [71] and to identify the best-suited evolutionary model by the Bayesian (BIC) and Akaike information criterion (AIC) [70]. Phylogenetic trees were visualized with Figtree (http://tree.bio.ed.ac.uk/software/figtree/) and iTOL [92].

### 2.5. Evolutionary Pressures on Acid Resistance Genes

Selective pressure on genes was determined by calculating the ratio of non-synonymous DNA substitutions (D_n_) to synonymous DNA substitutions (D_s_) in the coding region [93]. Individual genes (DNA and amino acid sequences) from all protein families were extracted using custom Perl scripts. Amino acid alignments were constructed using the MAFFT L-INSI iterative refinements option [65,66] and MUSCLE [67] and used as input for PAL2NAL [94] in conjunction with their nucleotide sequences to obtain the corresponding codon alignments for gene families. D_n_/D_s_ ratios were assigned for all possible pairwise comparisons within a protein family and calculated based on the codon alignments using the SeqinR package of the R project [95]. Mean D_n_/D_s_ ratios were assigned for individual gene families by averaging all pairwise ratios within each family. D_n_/D_s_ ratios of > 1 indicated beneficial mutations, and ratios of <1 indicated purifying selection [96].

### 2.6. Mapping Evolutionary Events

The inference of branch-site-specific events was made using the 16S rRNA gene tree of *Leptospirillum* genomes with *N. marina* Nb-295 as the outgroup. The presence and absence of genes related to acid resistance and associated genes located within the same genomic context were mapped onto each branch of the phylogenetic tree to model gene gain, loss, and modification events. Inference of evolutionary events was made using maximum parsimony criteria [97,98]. 

## 3. Results and Discussion

### 3.1. Genomic Features of Leptospirillum Genomes

Twelve publicly available genomes of the *Leptospirillum* genus were analyzed together with the genome of the neutrophile *N. marina*. *N. marina* is the closest phylogenetic relative of the Leptospirilli with available genomic data (Table 1). Five of the genomes were complete, while eight (including *N. marina*) were permanent drafts. “*Leptospirillum rubarum*”, *Leptospirillum* “5-way CG”, *Leptospirillum* “C-75”, and “*Leptospirillum ferrodiazotrophum*” were from metagenomic samples. The G + C content of *Leptospirillum* Group I, II, and III genomes were 50.1, 54.0, and 57.5%, respectively. Leptospirilli included both mesophiles and moderate thermophiles and grew between 40–43 °C. However, *L. ferrooxidans* C2-3, *L. ferriphilum* DSM 14647^T^, and *L. ferriphilum* Sp-Cl were mesophiles with growth temperatures between 30°C and 37°C. *Leptospirillum* sp. “UBA BS” (Group IV; NCBI Accession: PRJNA176861 [32]) was not included in this study because the genome did not meet the quality criteria of CheckM [60], exhibiting only 41% completeness with 38% contamination.

### 3.2. Phylogenetic Relatedness between Leptospirillum Species and Other Members of the Nitrospirae Phylum

**Figure 2:**
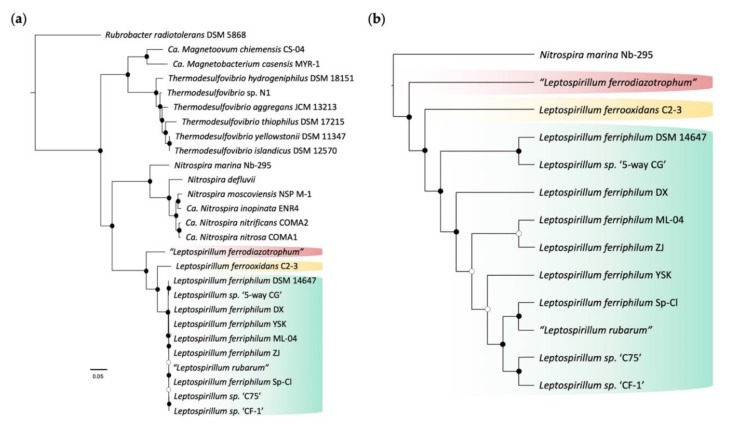
16S rRNA phylogeny and cladogram of *Leptospirillum* species with sequenced genomes used in this study. Clades belonging to *Leptospirillum* Groups I (yellow), II (green), and III (red) are indicated. (**a**) Phylogeny of the *Leptospirillum* genus inferred from 16S rRNA gene sequences. Filled circles at the nodes indicate bootstrap support ≥ 60% and open circles bootstrap support < 60%. The scale bar indicates the number of nucleotide substitutions per site. (**b**) Cladogram of the *Leptospirillum* genus derived from the 16S rRNA gene phylogenetic tree using *N. marina* as an outgroup. The cladogram shows the predicted branching order of the *Leptospirillum* genus. Bootstrap values are derived from Figure 2a. Color coding is the same as shown in Figure 2a.

A phylogenetic tree of the members of the Nitrospirae phylum was developed based on 16S rRNA gene sequences (Figure 2a). The tree was rooted using the validated *Rubrobacter radiotolerans*^T^ DSM 5868 as an outgroup. Three species of *Leptospirillum* can be distinguished in the phylogenetic tree: *L. ferrooxidans* (Group I), *L. ferriphilum* (Group II), and “*L. ferrodiazotrophum*” (Group III) with bootstrap support ≥ 60% (Figure 2a). This tree was consistent with published trees of *Leptospirillum* [51,101,105]. The phylogenetic branching points of the members of Group II had insufficient resolution to be separated (Figure 2a). Therefore, the phylogenetic tree was also presented as a cladogram (Figure 2b), showing their predicted branching order with bootstrap values. Based on the measurement of genetic distance, it was shown that *N. marina* Nb-295 was the closest extant relative with a sequenced genome to the *Leptospirillum* genus.

### 3.3. Gene Inventories

Genes with predicted or experimental evidence for functions related with first and second lines of defense to low pH environments were identified in the literature. A list of the genes used, their NCBI accession numbers, and their predicted features are provided in Supplementary Table S1.

### 3.4. First Line of Defense

#### 3.4.1. Membrane Potential and Potassium Transporters

*Kch*, potentially encoding a K^+^ channel protein, was identified in all *Leptospirillum* genomes, but not in *N. marina*. One possible explanation for this is that *kch* was incorporated into the *Leptospirillum* lineage by HGT after its separation from the *N. marina* lineage. Alternatively, *kch* was present in the last common ancestor and was lost in the *N. marina* lineage. Examination of the NCBI database using BLASTp showed that the best hits of the *Leptospirillum* Kch were with proteins of the *Acidithiobacillus* genus together with several other known acidophiles (Supplementary Figure S1). These microorganisms are frequently found in extremely acidic environments populated by *Leptospirillum* (e.g., [34,56,106,107]), strongly suggesting that they have shared *kch* via HGT. In addition, this result was consistent with the contention that *kch* is associated with acid resistance.

In *Leptospirillum* Group III, *kch* was adjacent to, but divergent from, a gene potentially encoding a phage holin-like protein that is involved in stress response and other functions (reviewed in [108]). In *Leptospirillum* Groups I and II, additional copies of the phage holin were located close to predicted genes encoding DNA uptake competence functions (ComEC). These are thought to be one of the major components involved in HGT (reviewed in [109]). In *Leptospirillum* Groups I and II, *kch* was clustered with two other predicted acid resistance genes, *slp*8 and *gad*A (discussed in Section 3.4.4 and Section 3.5.2, respectively). One possibility is that *kch*, *slp*8, and *gad*A entered the *Leptospirillum* genome by HGT, possibly via a phage mediated uptake mechanism. 

**Figure 3:**
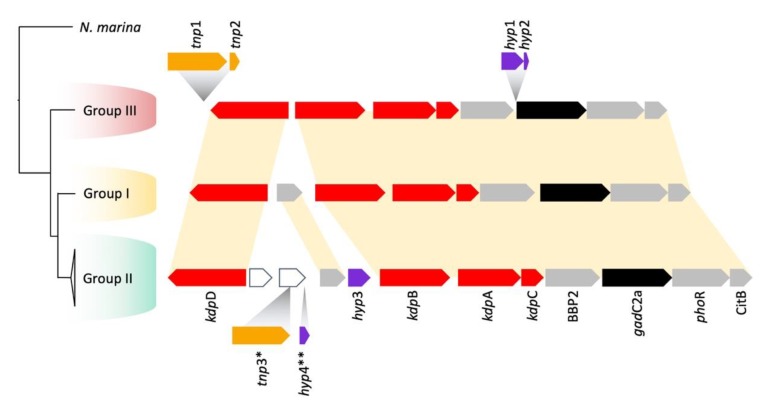
Phylogenetic distribution and genomic contexts of genes predicted for Kdp potassium uptake. *N. marina*, which lacks these genes, is included as an outgroup. Color coding of genes: red = Kdp genes, purple = orphan hypothetical genes, grey = additional genes whose genomic context is conserved, orange = predicted mobile elements and their remnants (* *tnp*3 inserted only in CF-1, ** *hyp*4 inserted only in the YSK strain), and black = *gad*C2A potentially involved proton export (see Section 3.5.2).

A *kdp*ABC gene cluster potentially encoding a potassium transporting Kdp P-type ATPase was found in all the *Leptospirillum* genomes (Figure 3). Downstream of the *kdp* cluster, there was a predicted BBP2 porin, a putative *gad*C2a permease, followed by another K^+^ sensing histidine kinase with a response regulator CitB. This cluster may be associated with K^+^ regulation. According to STRING analysis [77], this gene cluster was co-expressed in other species, suggesting that it was an operon. Some Group II genomes contained a predicted transposase (*tnp*3) associated with the insertion of two hypothetical genes. There was also an insertion of two hypothetical genes just upstream of *gad*C2a. The functions of the hypothetical genes remain unknown.

*Trk*A was identified in all *Leptospirillum* genomes and in the genome of *N. marina.* Comparative amino acid sequence analysis indicated that *Leptospirillum trk*A was found in a cluster that was separate from other *Nitrospira* (Supplementary Figure S2). An extremely large D_n_/D_s_ ratio was observed (~1) between *trk*A of *Leptospirillum* compared to other *Nitrospir*a, suggesting that it could have been vertically inherited from a likely neutrophilic common ancestor of *Leptospirillum* and other *Nitrospira* and then subjected to intense selective pressure to adapt to an acidic environment. Once adapted, it underwent a few additional changes, as shown by extremely low D_n_/D_s_ ratios (~0.05) within the *Leptospirillum* genus (Supplementary Figure S2).

#### 3.4.2. Spermidine Biosynthesis and Associated Genes

**Figure 4:**
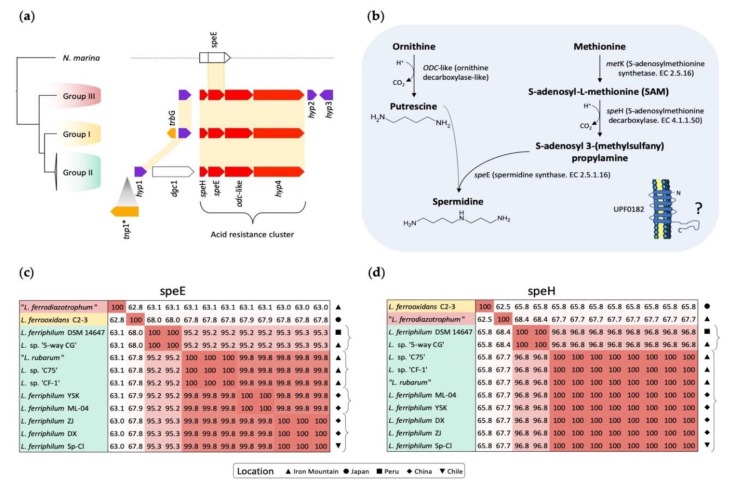
(**a**,**b**) Predicted spermidine biosynthesis genes and pathways of *Leptospirillum*. (**a**) Phylogenetic distribution and genomic contexts of spermidine biosynthesis genes. Color coding of genes: red = spermidine biosynthesis genes, purple = orphan hypothetical genes, orange = mobile elements and their remnants. * *tnp*1 was observed only in strain “CF-1” of Group II. (**b**) Predicted functions of the spermidine biosynthesis genes. (**c**,**d**) Heat maps of the nucleotide sequence identity of *spe*E (**c**) and *spe*H (**d**) in *Leptospirillum*. Red coloring indicates 100% nucleotide sequence identity, and numbers indicate the percent nucleotide identity.

The *Leptospirillum* genomes were searched for genes potentially encoding aliphatic polycation polyamines. No genes encoding for spermine or cadaverine biosynthesis were detected in any of the genomes. However, a conserved cluster of four genes potentially encoding spermidine biosynthesis was predicted in all three *Leptospirillum* groups, extending the earlier prediction of spermidine genes in in *L. ferriphilum* [13]. This cluster was not detected in *N. marina* (Figure 4 and Supplementary Figure S3). Three of the genes in the cluster were predicted to encode the biosynthesis of spermidine from S-adenosyl-L-methionine (SAM): *spe*H encoding S-adenosylmethionine decarboxylase, *spe*E encoding spermidine synthase, and an *odc*-like gene predicted to produce putrescine from ornithine (Figure 4a,b). The fourth gene (*hyp*4) encoded a conserved hypothetical protein UPF0182 found in many organisms in the same genomic context, but whose function remains unknown. UPF0182 was predicted to have a signal peptide for protein export and six transmembrane regions and was most likely to be an inner membrane protein.

An additional upstream gene, termed *dgc*1 (for diguanylate cyclase), was found only in Group II (Figure 4). This gene contained a predicted GGDEF domain and three associated GAF superfamily domains. In other organisms, the GGDEF domain is involved in cyclic diguanosine monophosphate turnover and the production of the secondary messenger C-di-GMP (reviewed in [110]). A functional GGDEF gene was reported in the extremely acidophilic genus *Acidithiobacillus*, although it was associated with the EAL rather than the GAF domains [111,112]. The secondary messenger C-di-GMP was implicated in the regulation of biofilm formation and other functions in many bacteria [113]. Spermidine has also been associated with both the formation and inhibition of biofilms in other bacteria [30,31].

All three *Leptospirillum* groups contained a hypothetical orphan gene (*hyp*1) upstream of the spermidine cluster, and Group III contained, in addition, two predicted hypothetical orphan genes downstream (*hyp*2 and *hyp*3; Figure 4a). Although *hyp*1 remains of unknown function, it has been identified in AMD community proteomes along with the full spermidine “acid resistance cluster” [52,55]. Transcripts for the spermidine genes have been detected in AMD community meta-transcriptomes [34].

Genes potentially involved in HGT and/or genome rearrangement were detected in the neighborhood of the spermidine cluster. These included a predicted P-type conjugative transfer protein TrbG with a signal sequence and lipoprotein signal and a TnpIS5-like sequence (Figure 4a).

Heat maps derived from alignments of DNA sequences of *spe*E and *spe*H in *Leptospirillum* Group II illustrated an important aspect of their evolution (Figure 4c,d). The DNA sequence of *spe*E was 100% conserved between some strains. For example, *spe*E of “*L. rubarum*”, *L.* sp. “C75”, and *L.* sp. “CF-1” from Iron Mountain, USA, shared 100% nucleotide sequence identity (Figure 4c). Inspection of the position of these strains in the phylogenetic cladogram (Figure 2b) suggested that *spe*E was inherited from their last common ancestor and its sequence subsequently maintained under strong selective pressure within the shared acidic environment of Iron Mountain. The close physical proximity of the strains could also facilitate genetic exchange and homologous recombination, contributing to the maintenance of DNA sequence similarity. The *spe*E of *L. ferriphilum* ZJ, DX, and Sp-Cl formed another cluster with 100% DNA sequence identity different from the Iron Mountain cluster (Figure 4c). Strains ZJ and DX were from China, whereas Sp-Cl was from Chile. In this case, close geographic proximity could not explain the sequence identity, and selective pressure resulting from a similar environment seemed more likely to account for the maintenance of sequence identity. A comparison of the nucleotide sequences of *spe*H showed 100% identity in strains of Group II *Leptospirillum* from Iron Mountain, China, and Chile, with two exceptions (Figure 4d). The exceptions were *L. ferriphilum* DSM 14647 (from Peru) and *L.* sp. ‘5-way CG’ (from Iron Mountain) that formed a second cluster with 100% sequence identity.

It was hypothesized that geographical proximity could potentially explain some of the evolutionary trajectories of *spe*E and *spe*H, perhaps by maintenance of sequence identity via homologous recombination [104]. However, adaptation of vertically inherited genes to similar acidic econiches was a more likely explanation for those strains not geographically juxtaposed (e.g., *L. ferriphilum* Sp-Cl and *L. ferriphilum* DSM 14647).

#### 3.4.3. Hopanoid Biosynthesis

**Figure 5:**
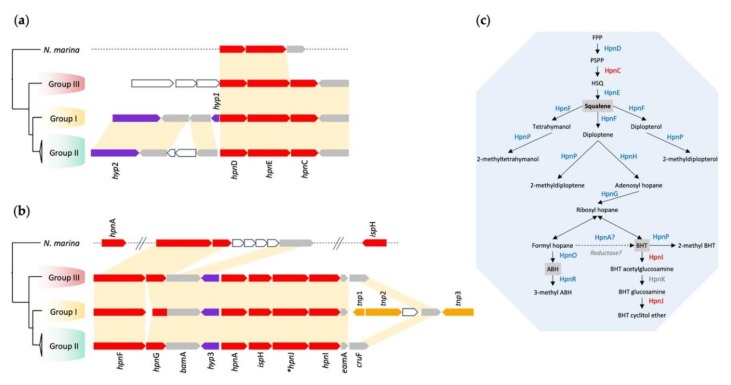
Genomic contexts for genes predicted to participate in (**a**) squalene and (**b**) hopanoid biosynthesis and bacteriohopanetetrol modification. Color coding of genes: red = squalene and hopanoid genes, grey = additional genes whose genomic context is conserved, purple = orphan hypothetical genes, orange = mobile elements and their remnants (*tnp*1-3), and white = other genes. * = *hpn*J1, *hpn*J2, and 3 are located in other genome locations. (**c**) Predicted pathways for squalene and hopanoid biosynthesis in *Leptospirillum* and *N. marina*. Blue lettering = biosynthetic steps encoded in both *Leptospirillum* and *N. marina*, and in red lettering, biosynthetic steps predicted only in *Leptospirillum*. Abbreviations: BHT = bacteriohopanetetrol, ABH = aminobacteriohopanetriol.

*Hpn*CDE, potentially encoding squalene and a core set of hopanoid biosynthesis genes (*hpn*FGAHROP), were identified in *Leptospirillum* and *N. marina* (Figure 5). All these genes showed considerable syntenic conservation within all *Leptospirillum* Groups and *N. marina*, suggesting that they were inherited from a common ancestor by vertical descent. On the other hand, *hpn*IJ, encoding enzymes that modify hopanoids to bacteriohopanetetrol cyclitol ether, were predicted only in the genomes of *Leptospirillum*. It was hypothesized that *hpn*IJ entered the genome of the ancestral *Leptospirillum* after its divergence from the other *Nitrospira*. Mobile elements (*tnp*1-3) were detected in the neighborhood of the *hpn* gene cluster in *Leptospirillum* Group I (Figure 5b), suggesting that HGT of the cluster into *Leptospirillum* may have occurred. *Hpn*IJ have been shown in *Burkholderia* to be involved in C_35_ extensions of hopanoids including bacteriohopanetetrol (BHT), BHT glucosamine, and BHT cyclitol ether, which are in turn involved in the response to environmental stress conditions including low pH [29].

#### 3.4.4. Slp Starvation Lipoprotein

Four copies of *slp* were identified in *N. marina* (termed *slp*s1-4) and an additional four copies were discovered in *Leptospirillum* (termed *slp*s5-8). Phylogenetic analysis of their amino acid sequences suggested that all eight copies were distinct and displayed different evolutionary trajectories (Supplementary Figure S4). It is possible that one or more of the *Leptospirillum slp*s were derived by vertical descent from the *slp*s of the inferred ancestor with *N. marina*. However, the long branch lengths derived from the phylogeny made it difficult to pin-point unambiguously the *Nitrospira* ancestral *slp* that gave rise to an ancestral *Leptospirillum slp*. 

The evolutionary trajectories of the *slp*s in some clades of *Leptospirillum* could be explained by the similarity of geographic location. For example, *slp*6 and *slp*7 were found in clades belonging mainly to Iron Mountain, USA, and exhibited 100% amino acid sequence identity (Supplementary Figure S5). However, geographical proximity could not explain all trajectories. For example, *slp*5 and *slp*7 of *L. ferriphilum* DSM 14647 from Peru had 100% amino acid sequence identity with the Iron Mountain, USA, clade (Supplementary Figure S5). Furthermore, *slp*8 of *L. ferriphilum* DSM 14647 from Peru had 100% amino acid sequence identity with *slp*8 from a Chinese location (*L. ferriphilum* ML-04) and with *Leptospirillum* sp. “5-way CG” from Iron Mountain, USA. We concluded that the inheritance pattern of the *slp*s could be explained either by geographic proximity or by adaptation to similar acidic econiches similar to that postulated for *spe*E and *spe*H, as described in Section 3.4.2

**Figure 6:**
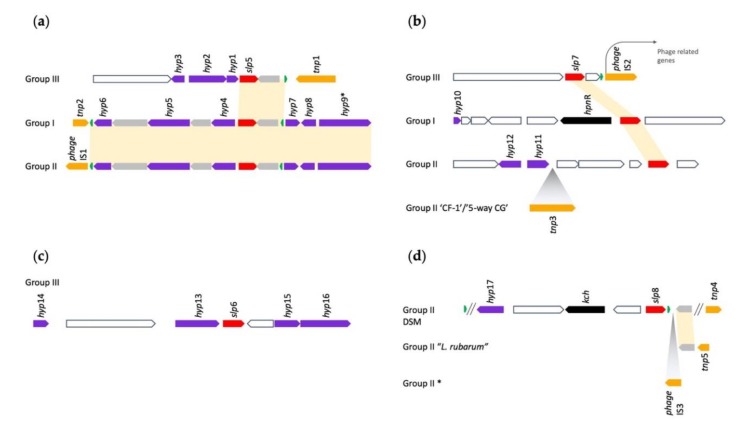
Genetic context of *slp* genes in *Leptospirillum*. (**a**) Genetic context for *slp*5 present in all *Leptospirillum;* (**b**) genetic context *slp*7 present in all *Leptospirillum*; (**c**) genetic context of *slp*6 present only in Group III; and (**d**) genetic context of *slp*8 present only Group II. Color coding of genes: red = *slp*, purple = orphan hypothetical gene, green = tRNA, gray = other conserved genes, orange = mobile elements, *tnp*1 (COG0675), *tnp*2 (PHA02517), *tnp*3 (pfam13701), *tnp*4 (COG3677), *tnp*5 (pfam13751), and *phage*IS, and black = *kch*, potentially involved in acid resistance (see Section 3.4.1). * = present in all strains of Group II except *L ferriphilum* DSM 14467 and “*L. rubarum*”.

Several deductions could be inferred from an inspection of the genomic contexts of the four *Leptospirillum slp*s (Figure 6): (i) All four *slp*s in each group displayed a different genomic context and, with the exception of *slp*5, also between species of the same group. The genomic context of *slp*5 was strongly conserved between Groups I and II and slightly conserved with Group III (Figure 6a). (ii) All four *slp*s were located near genes potentially encoding transposase-like functions, tRNAs, and phage-like genes, suggesting that they entered the genomes by HGT or underwent mobile element mediated rearrangement within these genomes. (iii) *slp*7 and *slp*8 were associated with *hpn*R and *kch*, respectively, genes potentially encoding other acid resistance functions (Figure 6b,d). (iv) All four *slp*s were associated with a number (18 in total) of orphan hypothetical genes with no known function. Given the context of these genes, they may be related to acid resistance or other stress-related functions and can be highlighted for future experimental analysis. Alternatively, they could be unidentified phage remnants.

Although the function(s) of the *Leptospirillum slp*s remain(s) unknown, all contain the lipobox motif that is characteristic of *slp*s in other organisms, at the end of a predicted signal peptide (Supplementary Figure S6) characteristic of *slp*s from other organisms [114]. The +2 position after the lipobox was proposed to be the main determinant for protein export such that if it had an Asp amino acid residue, then it was retained at the inner membrane (Supplementary Figure S6). An Asp was identified only in Slp5 from Group III, suggesting all the other proposed Slps might be exported to the outer membrane.

### 3.5. Second Line of Defense

#### 3.5.1. Proton Antiporters

One gene copy of the putative voltage gated ClC-type chloride/proton antiporter (ClcA) was identified in all *Leptospirillum* genomes, but not in *N. marina*. The genomic region around *clc*A was not conserved in any of the *Leptospirillum* Groups. However, each genomic context of *clc*A was associated with mobile elements or their remains. For example, a transposase DDE domain (cl26088) and a DNA recombinase Rci/bacteriophage Hp1-like integrase (cd00796) were in its neighborhood in Groups III and II. Cluster analysis of the ClcA amino acid sequences showed that the evolutionary trajectory of ClcA followed the pattern of the 16S rRNA phylogeny and was most likely inherited by vertical descent within the *Leptospirillum* groups (Supplementary Figure S7). We suggested that the mobile elements associated with *clc*A could have been involved in its chromosomal relocation in each group.

**Figure 7:**
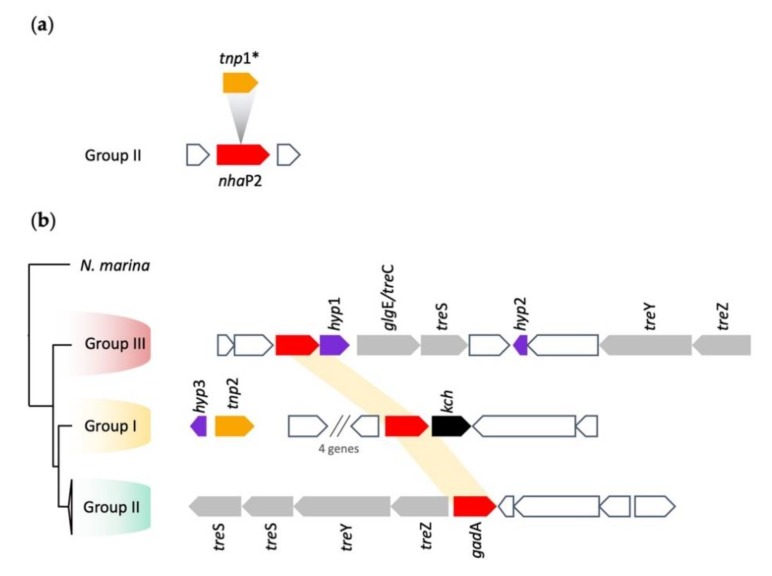
Gene context of (**a**) *nhaP*2 showing conservation in all strains of Group II. Color coding of genes: red = *nha*P2, orange mobile element *tnp*1 (transposase DDE_3; pfam13358) found only in strain ML-04. (**b**) Phylogenetic distribution of *gad*A in all *Leptospirillum* Groups. Color coding of genes: red = *gad*A, purple = orphan hypothetical genes, grey = conserved genes predicted to be involved in trehalose biosynthesis, orange = mobile element (*tnp*2 (PHA02517), black = *kch*, potentially involved in acid resistance (see Section 3.4.1), and white = other genes.

A second mechanism postulated to remove protons from the cytoplasm involved the NhaP sodium/proton antiporter for which two copies, termed *nha*P1 and 2, were identified only in *Leptospirillum* Group II. A cluster analysis of their amino acid sequences suggested they were members of two different families (Supplementary Figure S8). *Nha*P1 was located in a conserved genomic context in all members of Group II. Remnants of a number of transposases and integrases together with tRNA-Arg were detected in the gene neighborhood, suggesting that *nha*P1 was acquired by HGT in an ancestral *Leptospirillum* after its divergence with the last common ancestor. However, the conserved genomic context is consistent with the idea that it was inherited vertically within the different groups after the initial HGT event. A copy of *nha*P2 was found only in *L. ferriphilum* ML-04 (Group II). However, its sequence was interrupted by a transposase insertion (*tnp*1; Figure 7a). It was unlikely that *nha*P*2* was functional because the transposase insertion introduced stop codons in its reading frame and split the functional NhaP domain (COG0025) into two parts.

#### 3.5.2. Gad Decarboxylase

Four copies of the acid resistance amino acid permease (*gad*C) and one copy of the acid resistance amino acid decarboxylase (*gad*A) were identified in all *Leptospirillum*. In contrast, *N. marina* contained only one putative amino acid permease. The glutamate decarboxylase (*gad*A) did not show synteny in its genome context between the three groups (Figure 7b), and a cluster analysis suggested that the gene was introduced by HGT from Archaea (in separate events, as the top hits for the three groups were different; Supplementary Figure S9). In addition, four predicted *gad*C amino acid permeases encoding genes were identified in a separate genomic location to *gad*A with one of them in the gene context of potassium transporter Kdp system (Figure 3). Cluster analysis suggested one *gad*C copy was similar to the one found in the *N. marina* genome with three additional families present in the *Leptospirillum* genomes (Supplementary Figure S10).

*Gad*A (glutamate decarboxylase) in *Leptospirillum* Groups II and III was associated with a cluster of trehalose biosynthesis genes (Figure 7b). It has been shown that potassium, glutamate, and trehalose form part of a response to osmotic shock and acid stress in *E. coli* (reviewed in [115]), suggesting that a similar response was possible in *Leptospirillum* Groups II and III.

### 3.6. Model of Leptospirillum Acid Resistance

**Figure 8:**
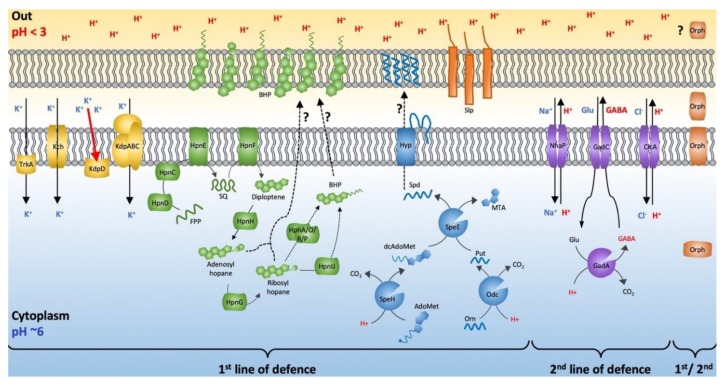
Model of acid resistance in *Leptospirillum*, categorized as either first line of defense involved in preventing the uptake of protons into the cell or second line of defense involved in neutralizing or expelling protons that enter the cell. Multiple hypothetical orphan genes (Orph) cluster with both first and second line of defense genes. The red arrow indicates that expression of KdpABC is activated by external K^+^ sensed by KdpD. The cytoplasm is conjectured to be about pH 6, as has been found in other extreme acidophiles, although this has not been experimentally determined in *Leptospirillum*.

A model of the *Leptospirillum* acid resistance systems, classified into first and second lines of defense mechanisms, is shown in Figure 8. Transcriptomic and proteomic analyses supported the relationship of the first line of defense genes with low pH adaptation in *Leptospirillum* [11,34,52,55,116]. Evidence also linked the expression of the KdpABC high-affinity potassium transport system and the HpnCDEFGIJHGRP and BamA hopanoid system to acid stress in AMD communities [34,52,55,116]. Environmental expression of genes involved in the second line of defense, such as the glutamate decarboxylase system *gad* and the Na+/H+ antiporter *nha*P, has been detected [34,116,117]. Clearly, additional experiments are required to explore the validity of the model. However, we posit that it provides a platform for helping to circumscribe future experimental directions.

Three genomic regions of *Leptospirillum* contained potassium transport system *kch* genes in proximity with other acid resistance genes, for example, *gad*C2 (Figure 3), *slp* (Figure 6), and *gad*A (Figure 7). This could allow their coordinated regulation. Each of these systems was associated with multiple mobile elements, suggesting that they could have entered the genome by HGT. Acid resistance genes were also found in close proximity to other stress responsive genes such as those involved in biofilm formation (Figure 4) and trehalose biosynthesis (Figure 7), potentially allowing coordination of genes involved in acid resistance and osmotic stress.

Whereas the inventory of potential mechanisms involved in first and second lines of acid resistance in *Leptospirillum* was quite extensive, they were by no means the only ones used by organisms for acid resistance. For example, in *Leptospirillum*, protons were hypothesized to be exported by the glutamate decarboxylase system, but evolutionary and mechanistically related systems such as ornithine decarboxylases have been implicated in acid stress responses in amateur acidophiles [86]. The extreme acidophile *Ferrovum*, belonging to the Betaproteobacteria class, has been hypothesized to use the Kef-type K^+^ transport system to help in maintaining a positive inside membrane potential and to utilize urease activity to neutralize its immediate environment [118]. External cellular capsule formation has been speculated to be involved in acid resistance in the Acidithiobacillia class [119]. None of these systems were predicted in *Leptospirillum*. Given that multiple acid resistance mechanisms were found in different Bacterial classes widely distributed in the Tree of Life, it was most likely that acid resistance evolved independently multiple times, perhaps aided by HGT. A similar conclusion has been made regarding the evolution of acid resistance in Archaea [120].

Genes encoding orphan hypothetical proteins located in genomic contexts associated with both first and second line of defense acid resistance genes may potentially encode unknown acid resistance mechanisms or functions that modify known acid resistance responses. Their bioinformatic prediction highlighted the need for experimental investigation into their functions.

### 3.7. Phylogenetic Distribution of Acid Resistance Genes and Their Inferred Evolutionary Trajectories

**Figure 9:**
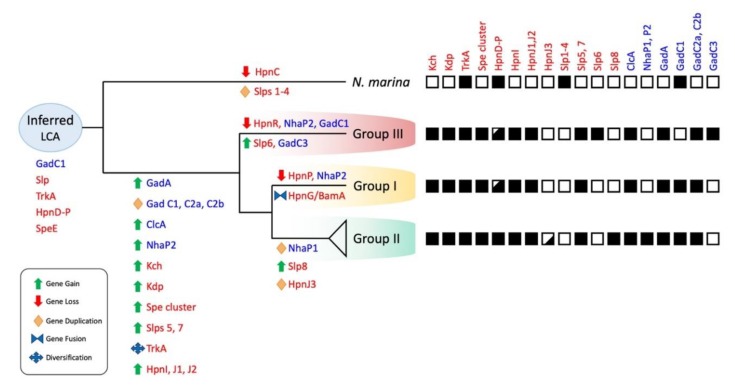
Inferred evolutionary reconstruction of the acid related genes of *Leptospirillum* with the main evolutionary events (gene gain/loss/fusion/duplication). Parsimony was used for the inferences. Names in red = first line of defense mechanisms and blue = second line of defense mechanisms. Black square = presence and white square = absence of mechanism. Black squares with white sections = mechanism is present in some, but not all, strains. LCA = last common ancestor.

Evolutionary events leading to acid resistance in the *Leptospirillum* genus were inferred using parsimony bioinformatic methods [121] and were mapped onto the branches of the phylogenetic tree of *Leptospirillum* (Figure 9).

Many of the predicted acid resistance genes were hypothesized to have been absent in the inferred last common ancestor of *Leptospirillum* and *N. marina*. These included genes encoding the K^+^ transporters Kch and Kdp, the four spermidine gene clusters, HpnIJ, ClcA, NhaP, and GadC2 and 3. It was proposed that they entered the *Leptospirillum* ancestral line by HGT via conjugation (e.g., the spermidine four-gene cluster), viruses (e.g., *slp*s7 and 8, *clc*A), and multiple examples involving transposases. HGT has been suggested to be a prevalent mechanism in genome evolution in a wide range of microorganisms [122].

With two exceptions, the donors of the HGT genes were difficult to trace, perhaps because the events occurred so long ago that molecular signals of the donors have been erased with the passage of time. The *Leptospirillum* Kch potassium transporter had several top BLASTp hits with other extreme acidophiles, including the *Acidithiobacillus* genus which shares its low pH environment. *Leptospirillum* is rooted deeper in the Tree of Life than *Acidithiobacillus* and therefore was probably ancestral to it, suggesting that the direction of transfer of Kch was from a *Leptospirillum* ancestor to an *Acidithiobacillus* ancestor. A second example of possible HGT donor identification lied in the comparison of the “*L. ferrodiazotrophum*” and “*L. rubarum*” glutamate decarboxylase acid resistance system with other members of the AMD community, suggesting that the genes were introduced by HGT into a *Leptospirillum* ancestor from an Archaeal *Ferroplasma* ancestor. 

Gene duplication and gene diversification events were identified using a combination of phylogenetic inference based on alignments of families (cluster analysis) [123] and calculations of D_n_/D_s_ [94,95,96]. Multiple examples of gene duplication events were detected. These included examples of potential vertical descent followed by gene duplication giving rise to paralogs (e.g., HpnJ1-3 in Group II). There were also many cases of gene duplications that were predicted to be xenologs, arising from HGT events. Xenologs were defined as a distinct form of horizontal gene transfer in which a gene was displaced by an ortholog from a different lineage [124], e.g., *slp*s5-8 in *Leptospirillum* replaced *slp*s present in the last common ancestor.

The relative timing of the events leading to the hypothesized transition of the ancestral *Leptospirillum* from a circumneutral or mildly acidic environment to a hyper-acidic one was difficult to assess. Based on what was known about acid stress response mechanisms of moderate (“amateur”) acidophiles (pH 3.5–6), it seemed likely that the first transition events involved the development of second line of defense mechanisms including proton expulsion mechanisms such as ClcA, NhaP, and Gad. However, it could not be ruled out that the mechanisms of the first line of defense were also involved in the early transition to very low pH environments. Some of these, such as hopanoids and spermidine, could have been gained initially to provide protection from other stresses such as oxidative stress or high temperature and subsequently adapted for low pH protection.

This paper focused on the potential mechanisms employed by *Leptospirillum* to thrive in extremely low pH environments. One of the major aspects of adaptation that was not investigated was how proteins fold and function in acid conditions. The cytoplasm was hypothesized to be around pH 6 or circumneutral, as was shown for other acidophiles, although this was not experimentally verified for *Leptospirillum*. If this assumption were correct, then only proteins or protein loops outside the periplasmic membrane would be exposed to low pH. Protein adaptations to low pH have been investigated in other acidophiles (e.g., [125,126]), but since no information was available for *Leptospirillum*, the model of its hypothesized transition from a neutral to an acid environment remains incomplete. Other major lacunae in our knowledge of the evolution of acidophilia in *Leptospirillum* were how changes in pH were sensed and transduced into gene regulation and how chaperones could be involved in maintaining protein integrity.

## Figures and Tables

**Table 1:** Properties of organisms and their genomes used in this study.

Genome	Size (Mb)	# Predicted Genes	G + C (%)	pH	Temp (°C)	Status ^1^	NCBI Accession ^2^	Geographical Origin	Refs
*Leptospirillum ferrooxidans* C2-3	2.5	2587	50.0	1.8	30	C	NC_017094.1	Mount Oyama, Miyake, Japan	[99]
*Leptospirillum ferriphilum* DSM 14647	2.6	2687	54.1	1.4–1.8	37	C	NZ_OBMB00000000.1	Enrichment culture, Peru	[13]
*Leptospirillum ferriphilum* ML-04	2.4	2475	54.6	2.5	40	C	NC_018649.1	Sulfide hot spring, Yunnan, China	[46]
*Leptospirillum ferriphilum* YSK	2.3	2361	54.1	1.6	40	C	NZ_CP007243.1	Dexing copper mine, JiangXi, China	[100]
*Leptospirillum ferriphilum* DX	2.3	2381	54.5	1.5	40	D	NZ_MPOJ00000000.1	Dexing copper mine, JiangXi, China	[101]
*Leptospirillum ferriphilum* ZJ	2.3	2449	54.7	1.5	40	D	NZ_MPOK00000000.1	Zijinshan copper mine, Fujian, China	[101]
*Leptospirillum ferriphilum* Sp-Cl	2.4	2552	54.4	1.5	37	D	NZ_LGSH00000000.1	Spence mine, Chile	[59]
*Leptospirillum* sp. “CF-1”	2.7	2731	54.6	1.6–1.7	40	C	NZ_CP012147.1	Iron Mountain, CA, USA	[102]
*Leptospirillum* sp. “C75”	2.6	2528	54.4	0.7–1.2	40–43	D	GCF_000262365.1	Iron Mountain, CA, USA	[103]
*Leptospirillum* sp. “5-way CG”	2.7	2633	51.5	0.8	42	D	DS995259.1–DS995275.1	Iron Mountain, CA, USA	[104]
*“Leptospirillum rubarum”*	2.6	2654	54.7	1.1	41	D	GCA_000205145.2	Iron Mountain, CA, USA	[52]
*“Leptospirillum ferrodiazotrophum”*	2.8	2727	57.5	1.1	41	D	GG693851.1–GG693892.1	Iron Mountain, CA, USA	[52]
*Nitrospira marina* Nb-295	4.6	4276	50	6.4–7.5	30	D	2596583682	Gulf of Maine, USA	^‡^

^1^ Sequence status where C: Complete, D: Draft. ^2^ NCBI RefSeq accession numbers are provided; if not available, then NCBI RefSeq assembly IDs are provided. ^‡^ IMG genome ID (https://img.jgi.doe.gov/). pH and temperature for organismal growth come from culture experiments or from in situ environmental measurements.

## Supplementary Materials

The following are available online at https://www.mdpi.com/2073-4425/11/4/389/s1, **Figure S1**: Unrooted phylogenetic tree constructed from the predicted amino acid sequences of Kch in the *Leptospirillum* genus. **Figure S2**. (**a**) Unrooted phylogenetic tree constructed from the predicted amino acid sequences from TrkA in the *Leptospirillum* genus. (**b**) D_n_/D_s_ box plots for TrkA. **Figure S3**. Unrooted phylogenetic tree constructed from the predicted amino acid sequences of SpeE in the *Leptospirillum* genus. **Figure S4**. Phylogenetic tree for *slp* gene copies (in different background highlighted colors) from the *Leptospirillum* genus. **Figure S5**. Heatmap of amino acid identity sequences of *slp* genes for the *Leptospirillum* genus. **Figure S6**. Multiple sequence alignment of *N. marina* and *Leptospirillum slp* gene sequences including a WebLogos plot of the *slp* lipobox. **Figure S7**. Unrooted phylogenetic tree constructed from the predicted amino acid sequences from ClcA in the *Leptospirillum* genus. **Figure S8**. Unrooted phylogenetic tree constructed from the predicted amino acid sequences from NhaP1 and 2 in *Leptospirillum* Group II. **Figure S9**. Unrooted phylogenetic tree constructed from the predicted amino acid sequences from GadA in the *Leptospirillum* genus. **Figure S10**. Unrooted phylogenetic tree constructed from the predicted amino acid sequences from GadC in the *Leptospirillum* genus. **Table S1**. 16S rRNA gene sequences used for the phylogenetic tree for the 26 Nitrospirae used in the study; best hit IDs used in the clustering analysis; IDs for the acid resistance genes; predicted locations of the hypothetical genes included in the study and the names and accessions for acid resistant genes not found in Leptospirillum.
