# Supplementary material for: Evolution of Predicted Acid Resistance Mechanisms in the Extremely Acidophilic Leptospirillum Genus
Source: Genes (Basel). 2020 Apr 3;11(4):389. doi: 10.3390/genes11040389 (PMC7231039; doi:10.3390/genes11040389)

Article

Evolution of Predicted Acid Resistance Mechanisms in the Extremely Acidophilic *Leptospirillum* Genus

Eva Vergara ^1^, Gonzalo Neira ^1^, Carolina González ^1,2^, Diego Cortez ^1^, Mark Dopson ^3^ and
David S. Holmes ^1,2,4,^*

^1^ Center for Bioinformatics and Genome Biology, Fundación Ciencia & Vida, Santiago 7780272, Chile; evamarilyn.vj@gmail.com (E.V.); [gonzalo.neira@ug.uchile.cl](mailto:gonzalo.neira@ug.uchile.cl) (G.N.); [carola.mgr@gmail.com](mailto:carola.mgr@gmail.com) (C.G.); [diegonahuel8@gmail.com](mailto:diegonahuel8@gmail.com) (D.C);

^2^ Centro de Genómica y Bioinformática, Facultad de Ciencias, Universidad Mayor, Santiago 8580745, Chile

^3^ Centre for Ecology and Evolution in Microbial Model Systems, Linnaeus University, Kalmar SE-391 82, Sweden; mark.dopson@lnu.se

^4^ Universidad San Sebastian, Santiago 7510156, Chile

**Supplementary Figure S1.** Unrooted phylogenetic tree constructed from the predicted amino acid sequences of Kch in the *Leptospirillum* genus and their best hits in the NCBI database which are mostly acidophiles (including the Acidithiobacilli) with the exception of SNU88298.1 and WP_106448183.1, which are neutrophiles. In addition, the context is shown for *kch* in Group III next to a predicted phage holin gene (Phage_holin_4_2, pfam04020). This juxtaposition only exists in Group III. Corresponding accession numbers and organism names are provided in Supplementary Table S1. Bar represent 0.1 amino acid substitution per site.


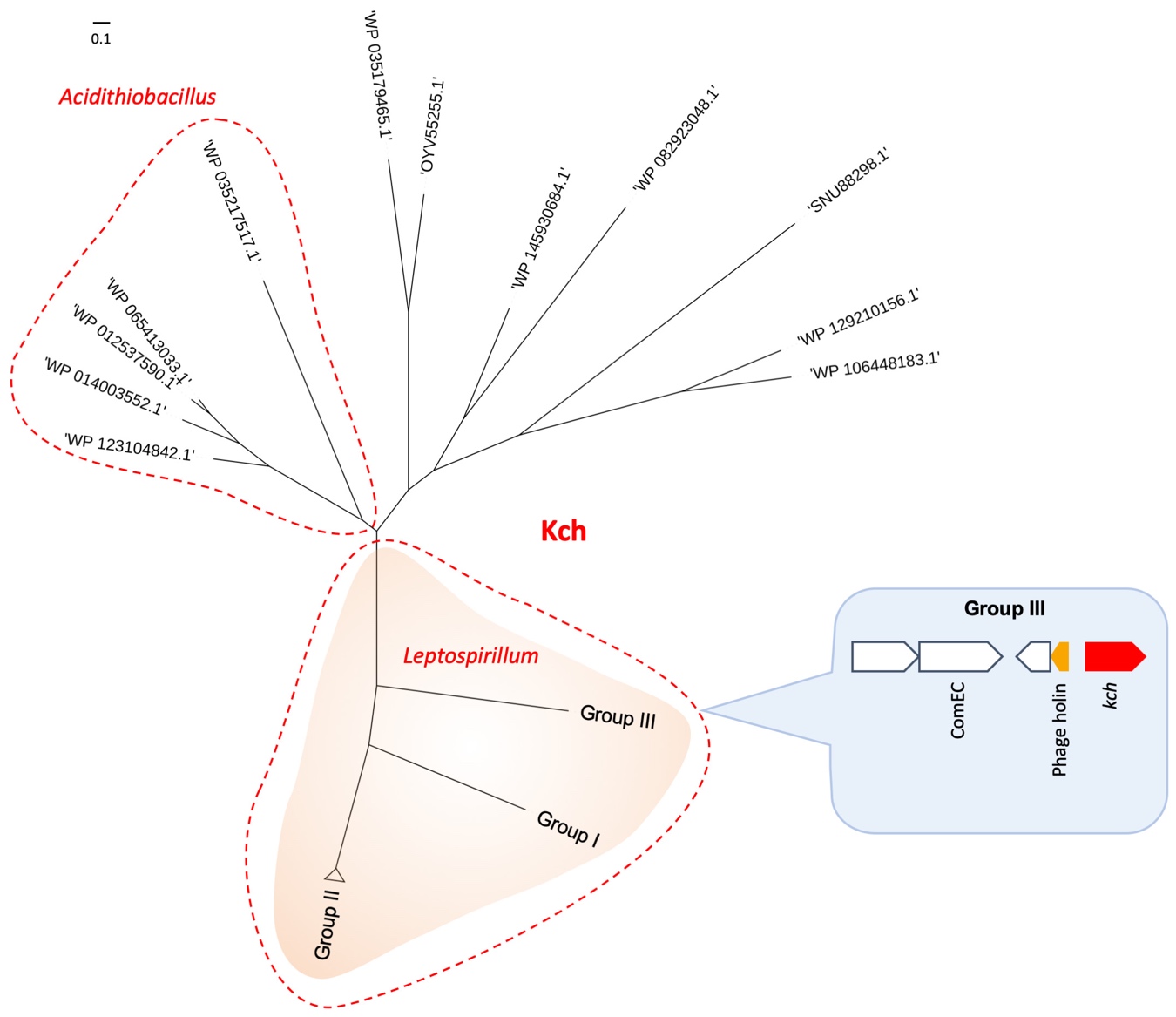


**Supplementary Figure S2.** (**a**) Unrooted phylogenetic tree constructed from the predicted amino acid sequences from TrkA in the *Leptospirillum* genus plus the outgroup and their best hits in the NCBI database. *Leptospirillum* TrkA clusters apart from the Nitrospira clade showing its evolutionary diversification. Corresponding accession numbers are provided in Supplementary Table S1. (**b**) D_n_/D_s_ box plots for TrkA, showing D_n_/D_s_ ratios of *trk*A genes of *Leptospirillum* genus (mean 0.05) and D_n_/D_s_ ratios of *Leptospirillum* genus with *N. marina* outgroup (mean 1.0) indicating the accumulation of a substantial number of mutations between *Leptospirillum* and *N. marina*, presumably due to selection for activity in an acidic environment, versus stabilization of the TrkA sequence within the *Leptospirillum* genus. Bar represent 0.1 amino acid substitutions per site.


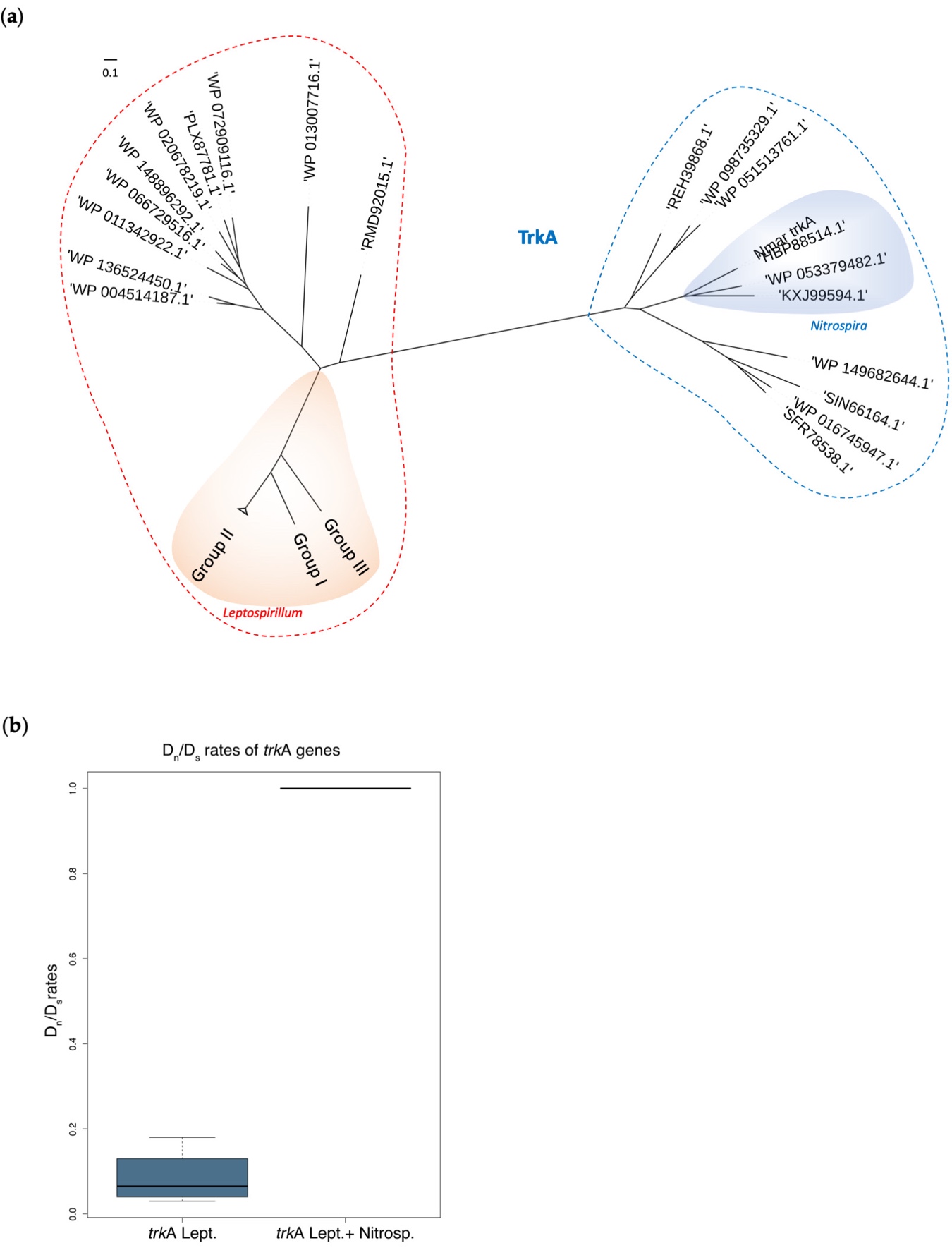

**Supplementary Figure S3.** Unrooted phylogenetic tree constructed from the predicted amino acid sequences of SpeE in the *Leptospirillum* genus plus the *N. marina* outgroup and their best hits in the NCBI database. Corresponding accession numbers are provided in Supplementary Table S1. SpeE from *Leptospirillum* forms a cohesive clade distinct from SpeE in *N. marina*. Bar represent 1.0 amino acid substitutions per site.


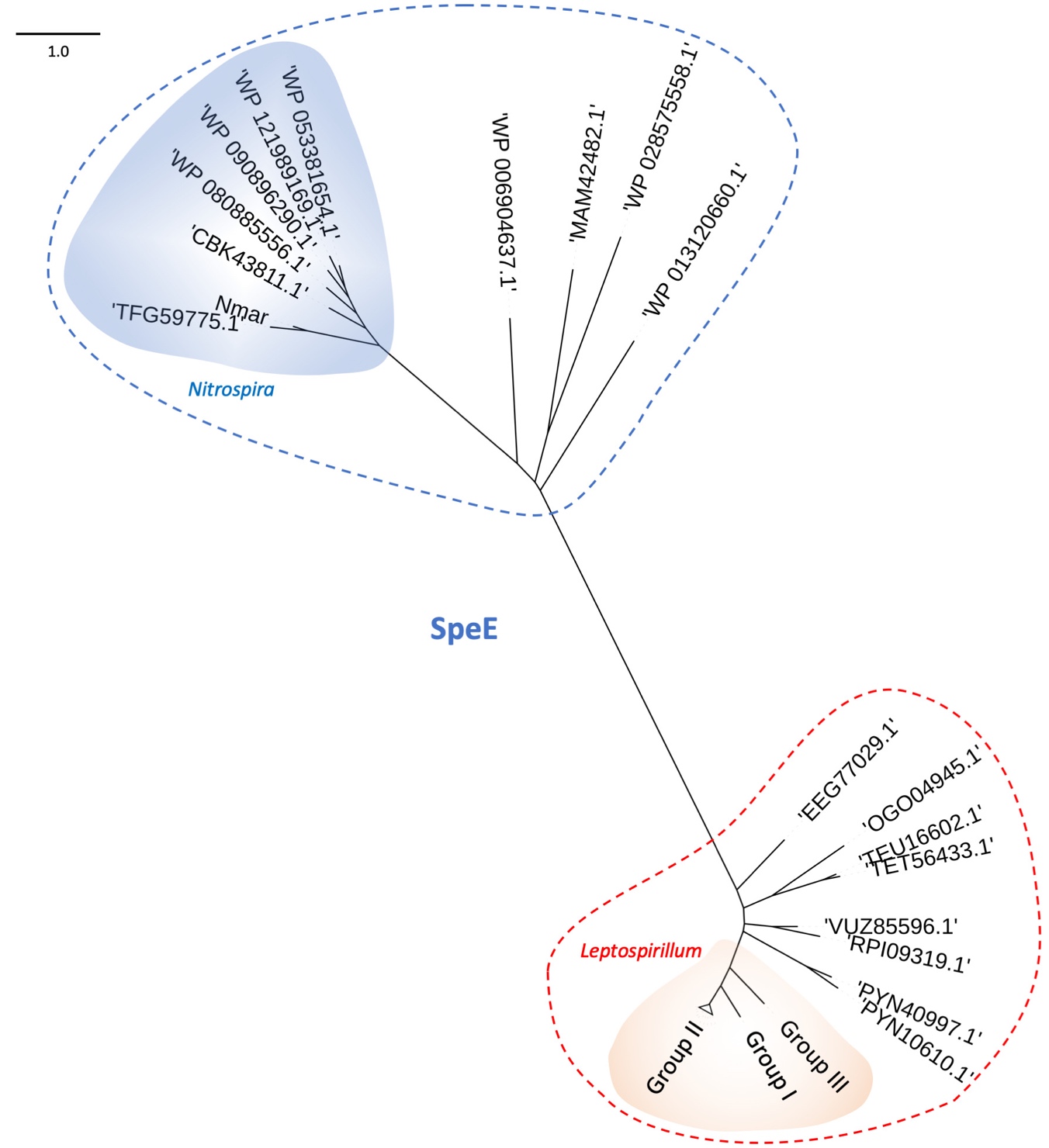


**Supplementary Figure S4.** Phylogenetic tree for *slp* gene copies (in different background highlighted colors) from the *Leptospirillum* genus using the copies found on *N. marina* as an outgroup. The geographic location for each genome is designated with the shapes next to each name. Scale bar, 0.2 substitutions per amino acidic position.


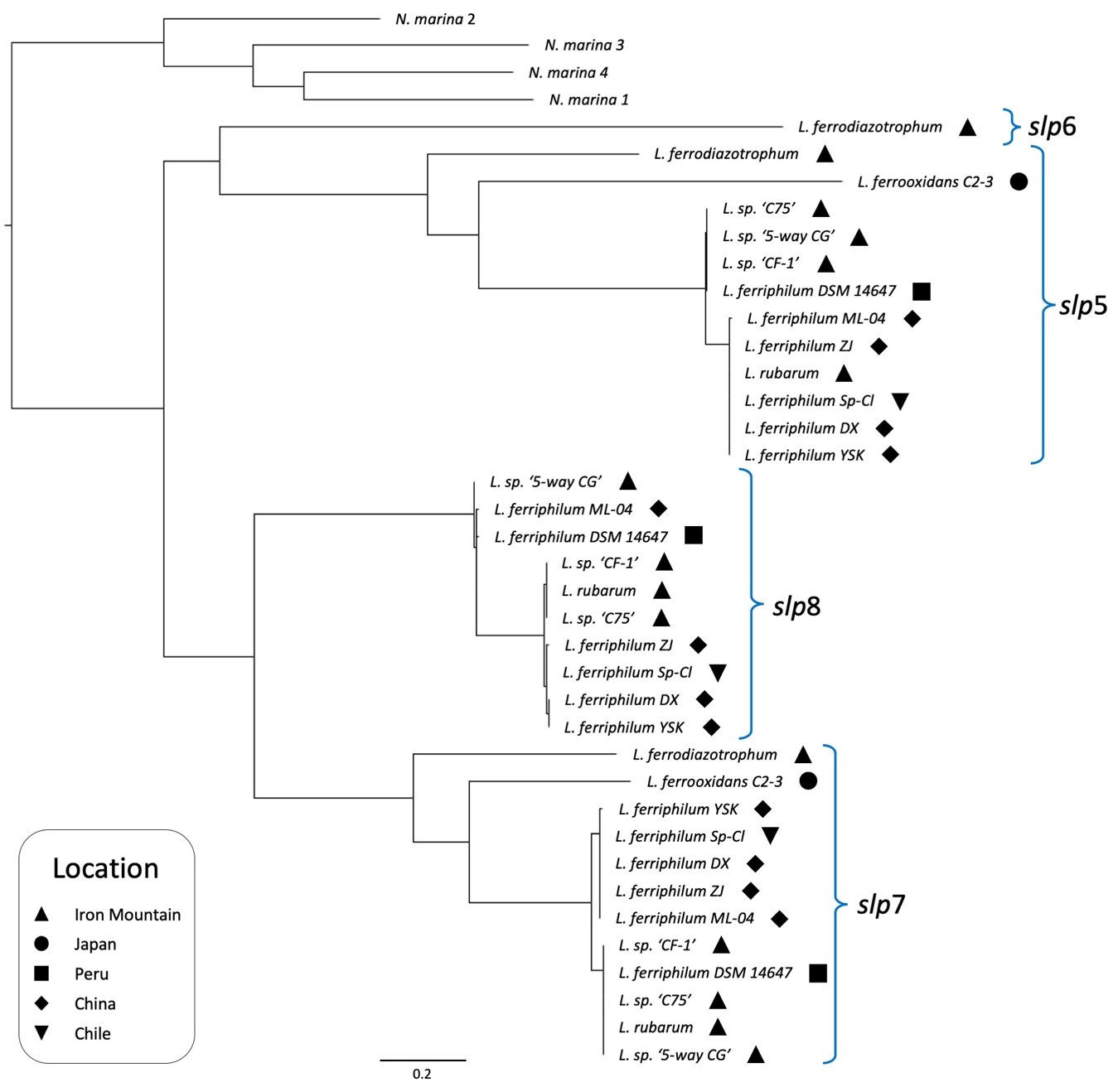


**Supplementary Figure S5.** Heatmap of amino acid identity sequences of *slp* genes for the *Leptospirillum* genus plus the *N. marina* outgroup.


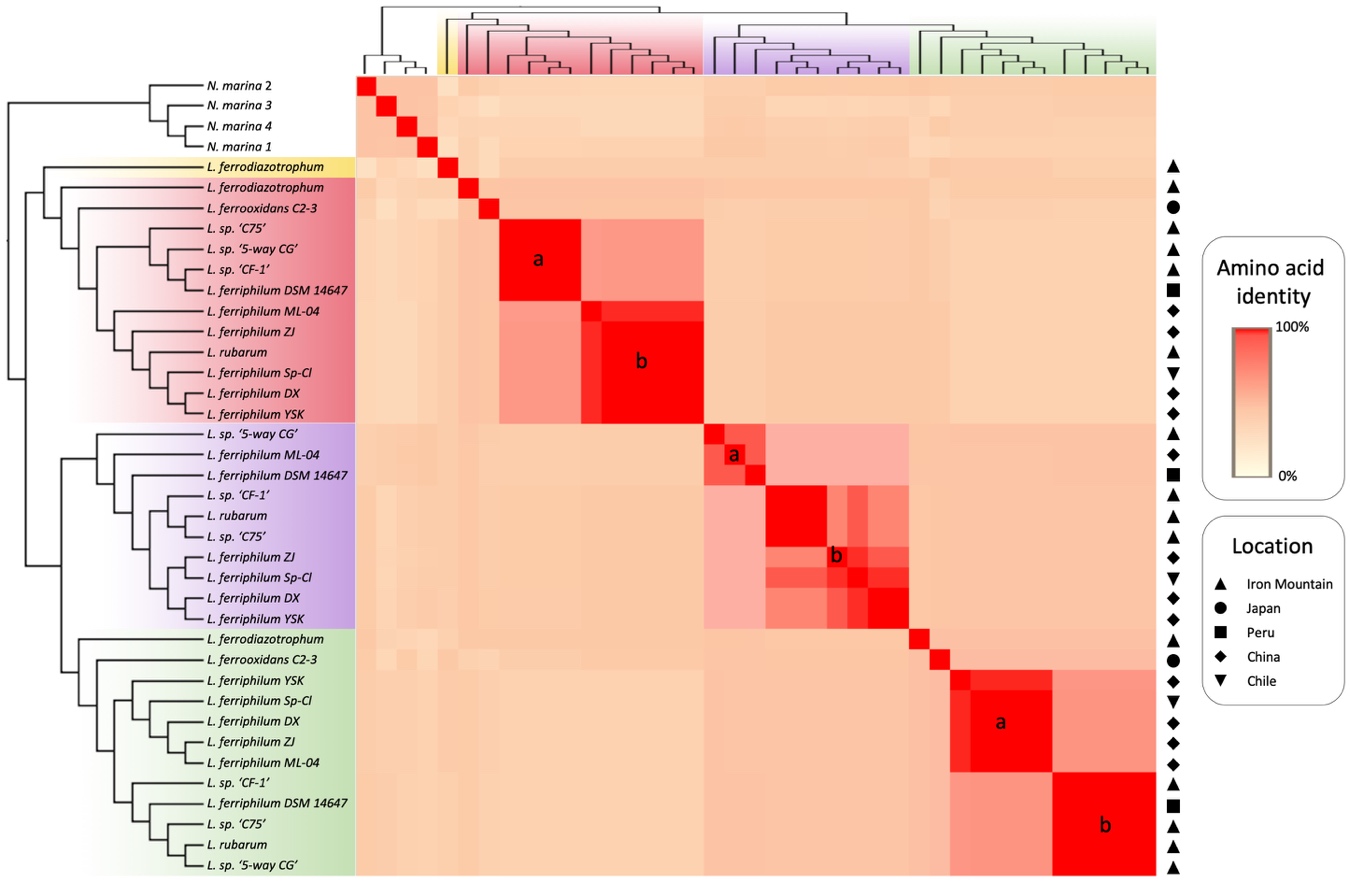


**Supplementary Figure S6.** Multiple sequence alignment of *N. marina* and *Leptospirillum* *slp* gene sequences including a WebLogos plot of the *slp* lipobox.

**
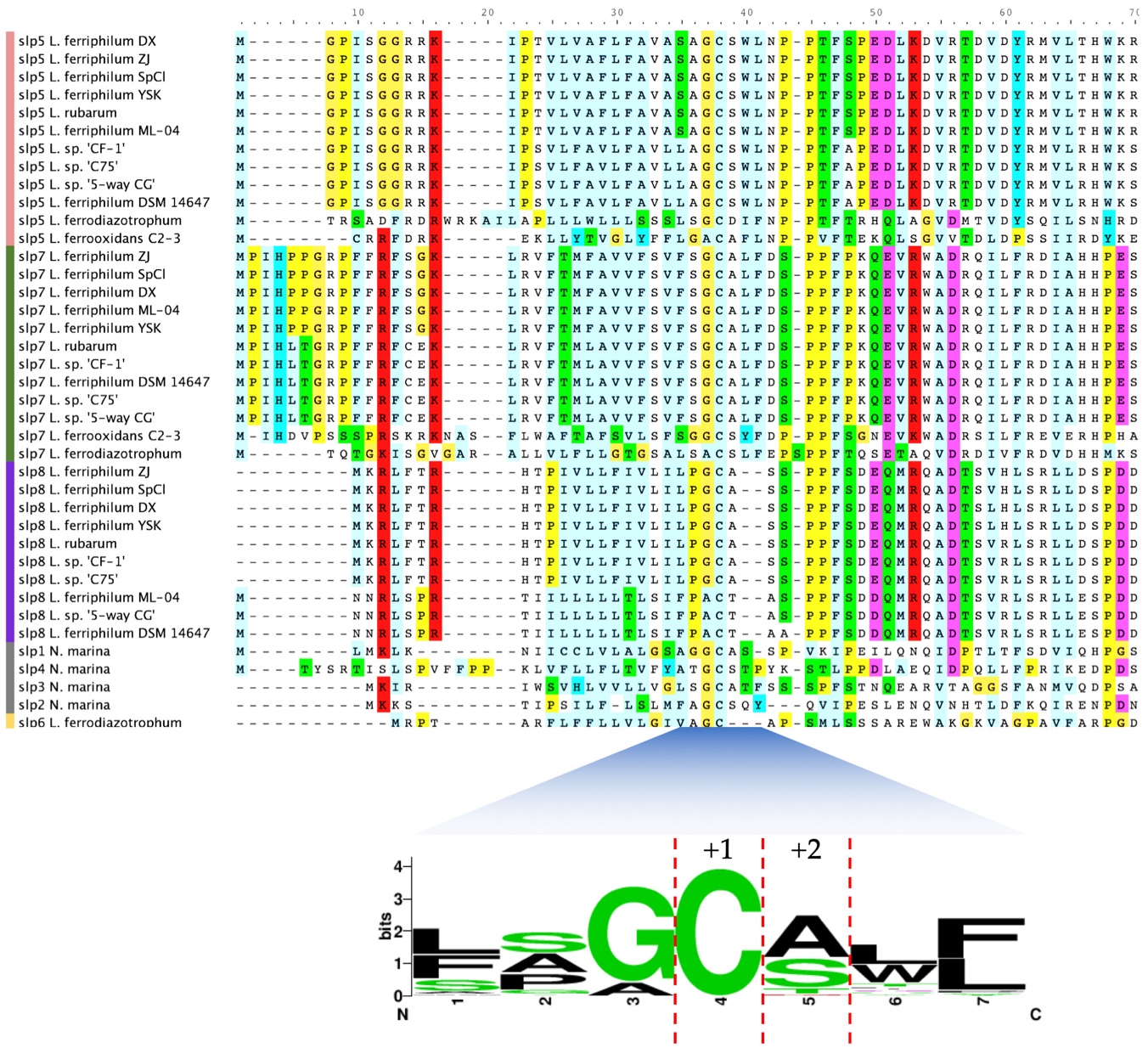
**

**Supplementary Figure S7.** Unrooted phylogenetic tree constructed from the predicted amino acid sequences from ClcA in the *Leptospirillum* genus and their best hits in the NCBI database. Corresponding accession numbers are provided in Supplementary Table S1. ClcA from *Leptospirillum* forms a distinct cluster with sequence similarity to ClcA from Archaea, Acidobacteria and Actinaobacteria*.* Scale bar, 0.1 substitutions per amino acidic position.


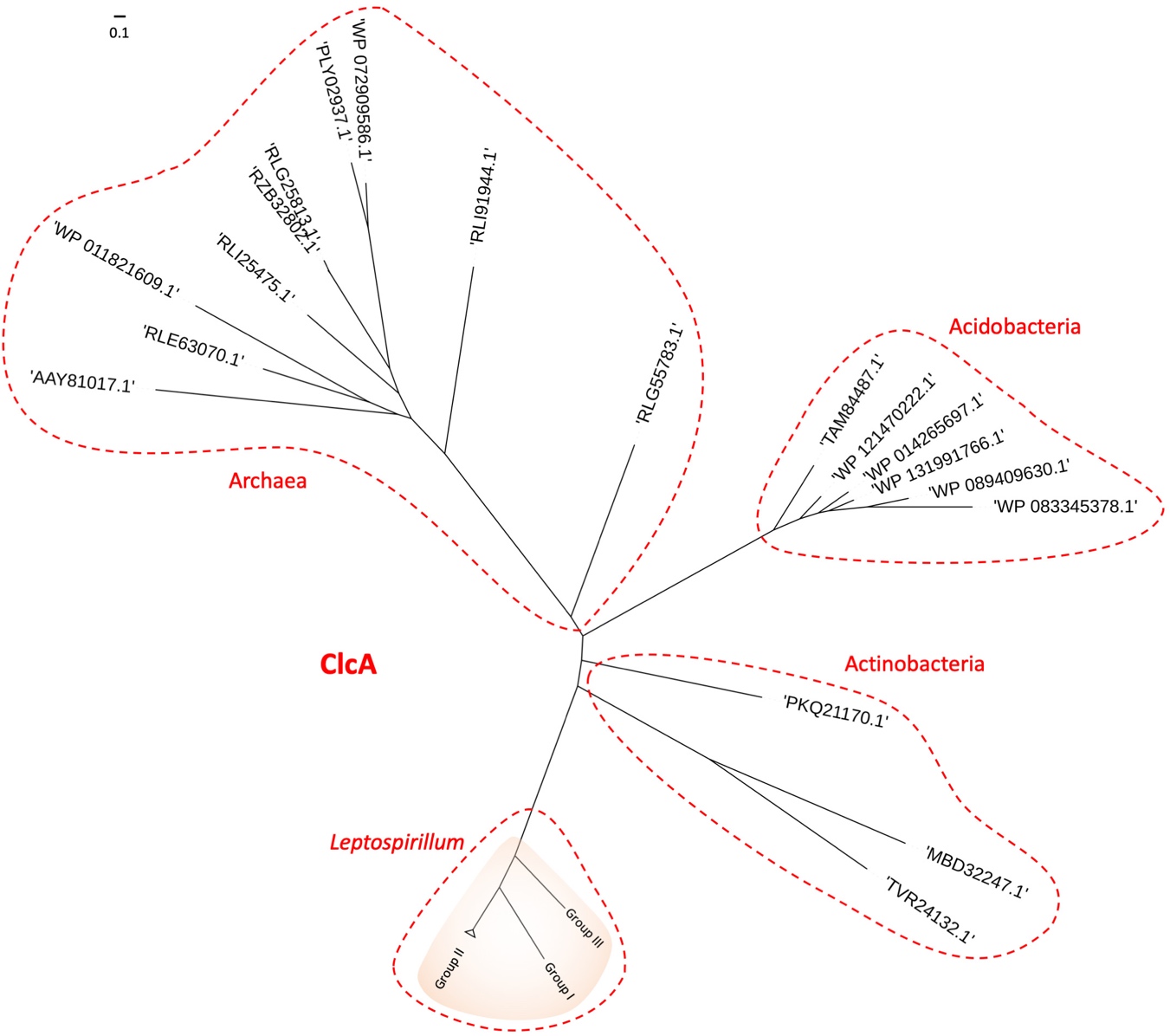


**Supplementary Figure S8.** Unrooted phylogenetic tree constructed from the predicted amino acid sequences from NhaP1 and 2 in *Leptospirillum* Group II and their best hits in the NCBI database. Corresponding accession numbers are provided in Supplementary Table S1. NhaP1 and NhaP2 form distinct clusters. Bar represent 1.0 amino acid substitutions per site.


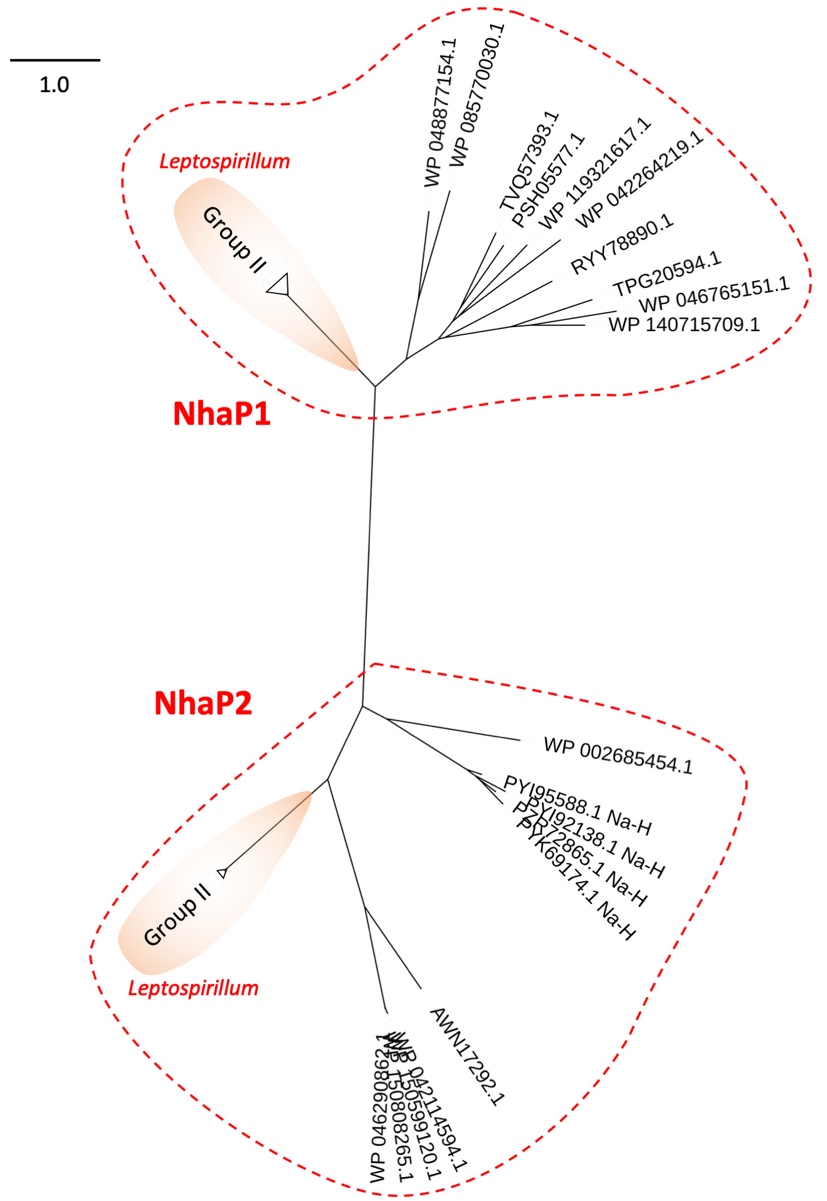


**Supplementary Figure S9.** Unrooted phylogenetic tree constructed from the predicted amino acid sequences from GadA in the *Leptospirillum* genus and their best hits in the NCBI database. Corresponding accession numbers are provided in Supplementary Table S1. GadA has two forms, one that is found in *Leptospirillum* Groups I and II, and a different copy that is found in Group III. Bar represent 0.1 amino acid substitutions per site.


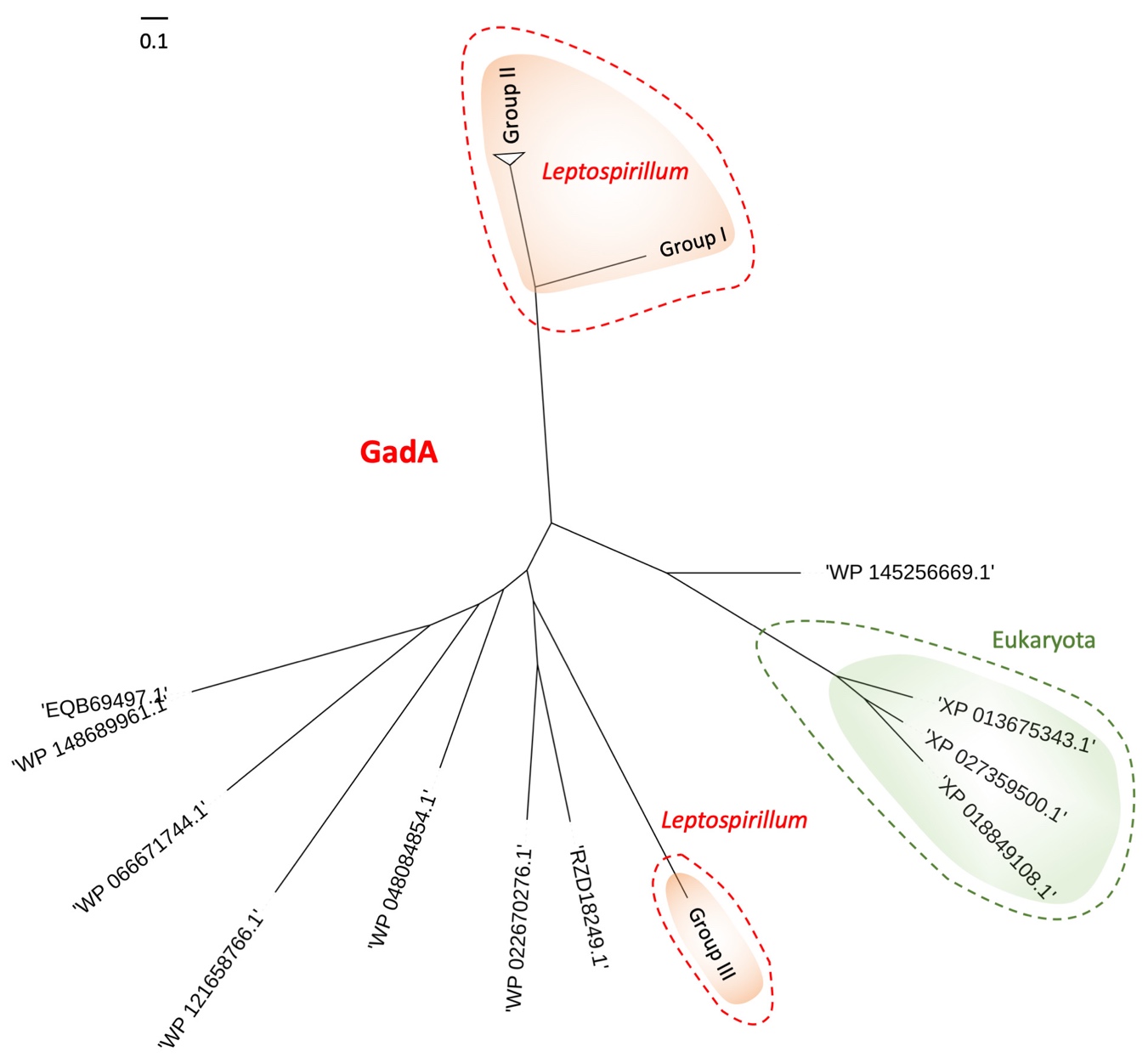


**Supplementary Figure S10.** Unrooted phylogenetic tree constructed from the predicted amino acid sequences from GadC in the *Leptospirillum* genus and their best hits in the NCBI database. Corresponding accession numbers are provided in Supplementary Table S1. GadC1 is phylogenetically related to *N. marina* and other *Nitrospira*, whereas GadC2 and GadC3 cluster separately. Bar represent 1.0 amino acid substitutions per site.


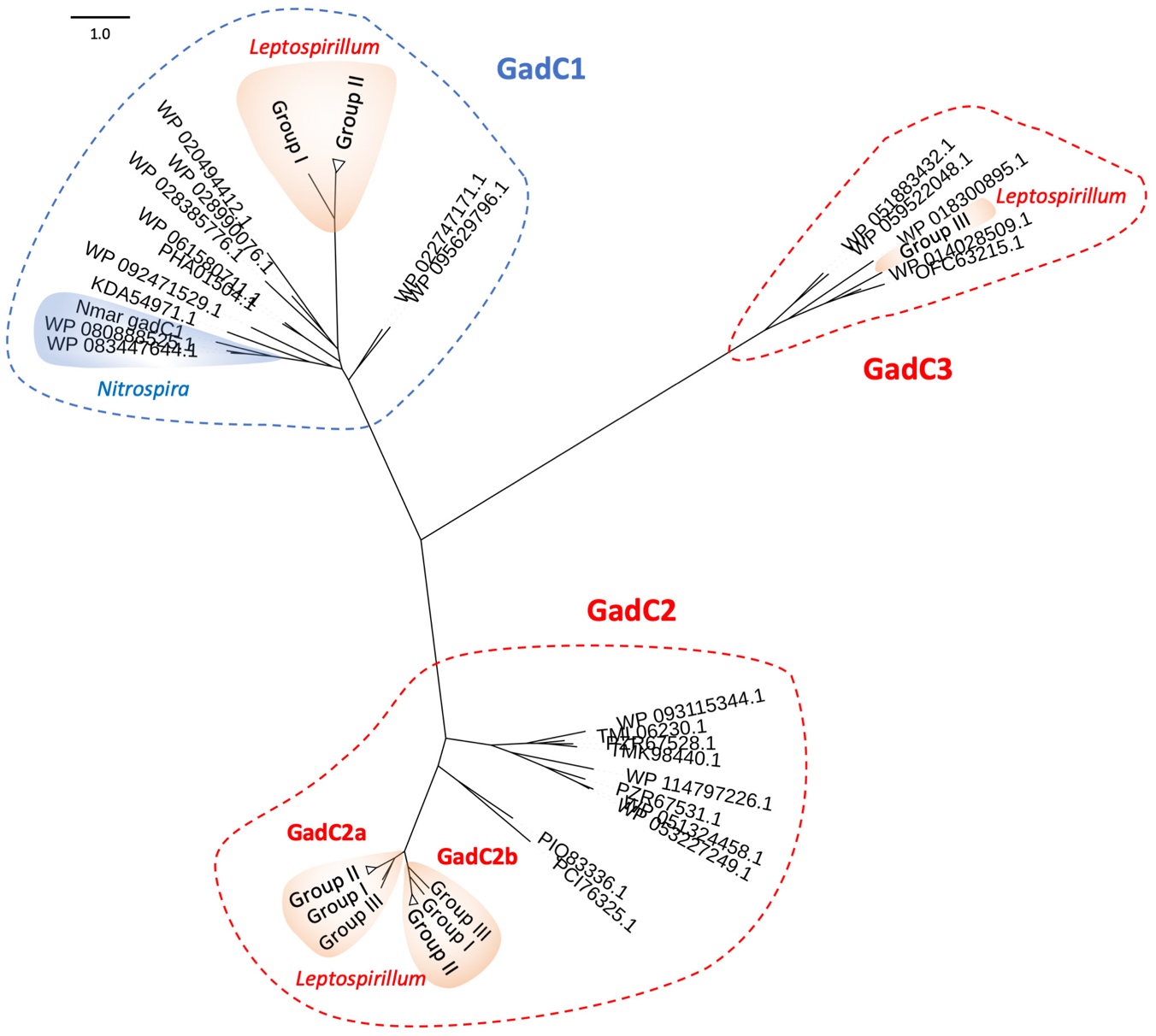

Supplement: Supplementary file 1 [file genes-11-00389-s001.zip › Supplementary_files_proofed.docx]
